# Incorporation of alternative amino acids into cyanophycin by different cyanophycin synthetases heterologously expressed in *Corynebacterium glutamicum*

**DOI:** 10.1186/s13568-021-01217-5

**Published:** 2021-04-15

**Authors:** Ramona Wördemann, Lars Wiefel, Volker F. Wendisch, Alexander Steinbüchel

**Affiliations:** 1 Institut für Molekulare Mikrobiologie und Biotechnologie (IMMB), Westfälische Wilhelms-Universität Münster, Corrensstraße 3, 48149 Münster, Germany; 2 Faculty of Biology & CeBiTec, Bielefeld University, Universitätsstraße 25, 33615 Bielefeld, Germany; 3 Environmental Science Department, King Abdulaziz University, Jeddah, Saudi Arabia

**Keywords:** Alternative amino acids, *Corynebacterium glutamicum*, Cyanophycin, Lysine, Ornithine

## Abstract

Cyanophycin (multi-l-arginyl-poly-l-aspartic acid; also known as cyanophycin grana peptide [CGP]) is a biopolymer that could be used in various fields, for example, as a potential precursor for the synthesis of polyaspartic acid or for the production of CGP-derived dipeptides. To extend the applications of this polymer, it is therefore of interest to synthesize CGP with different compositions. A recent re-evaluation of the CGP synthesis in *C. glutamicum* has shown that *C. glutamicum* is a potentially interesting microorganism for CGP synthesis with a high content of alternative amino acids. This study shows that the amount of alternative amino acids can be increased by using mutants of *C. glutamicum* with altered amino acid biosynthesis. With the DM1729 mutant, the lysine content in the polymer could be increased up to 33.5 mol%. Furthermore, an ornithine content of up to 12.6 mol% was achieved with ORN2(P_*gdh*4_). How much water-soluble or insoluble CGP is synthesized is strongly related to the used cyanophycin synthetase. CphA_Dh_ synthesizes soluble CGP exclusively. However, soluble CGP could also be isolated from cells expressing CphA_6308_Δ1 or CphA_6308_Δ1_C595S in addition to insoluble CGP in all examined strains. The point mutation in CphA_6308_Δ1_C595S partially resulted in a higher lysine content. In addition, the CGP content could be increased to 36% of the cell dry weight under optimizing growth conditions in *C. glutamicum* ATCC13032. All known alternative major amino acids for CGP synthesis (lysine, ornithine, citrulline, and glutamic acid) could be incorporated into CGP in *C. glutamicum*.

**Supplementary Information:**

The online version contains supplementary material available at 10.1186/s13568-021-01217-5.

## Introduction

The polyamide cyanophycin is typically composed of poly(aspartic acid) as a backbone to which arginine residues are attached; it is also referred to as multi-l-arginyl-poly-l-aspartic acid or as cyanophycin grana peptide (CGP). CGP is non-ribosomal polypeptide synthesised by cyanophycin synthetases (CphA, EC 6.3.2.29/EC 6.3.2.30). During heterologous production of CGP, other alternative amino acids can also be incorporated into the polymer in addition to aspartic acid and arginine. Instead of arginine, larger amounts of lysine, citrulline or ornithine and several others were also incorporated (Berg et al. 2000; Steinle et al. 2009; Wiefel et al. 2011). Glutamic acid can also be incorporated into the backbone as an alternative to aspartic acid (Wiefel et al. 2019a and references cited therein). In addition, biochemically incorporated constituents can be modified enzymatically or chemically (Frommeyer et al. 2014; Wiefel and Steinbüchel 2016, Wiefel 2019b). These variations of the polymer can change the solubility behavior of the polymer (Wiefel and Steinbüchel 2014). Variations in CGP are especially interesting to extend the possible field of applications of CGP. For example, CGP-derived dipeptides can be used as food supplements or animal feed additives or may have medical and cosmetic applications (Sallam and Steinbüchel 2010). The dipeptides are produced by enzymatic digestion of CGP employing cyanophycinase (intracellular CphB or extracellular CphE; EC 3.4.15.6), usually producing Asp-Arg dipeptides and related oligopeptides (Sallam et al. 2009).

*Corynebacterium* *glutamicum* in particular could be an interesting candidate for the production of CGP with a high content of alternative amino acids, as this bacterium is traditionally used for the production of amino acids on an industrial scale (Wendisch 2020). *C. glutamicum* is best known for the production of glutamate and lysine, both of which can be used for the synthesis of CGP. However, the other amino acids, which can be used for the synthesis of CGP, can also be produced in larger quantities by *C. glutamicum*. On the other hand, the thick and rigid cell wall of *C. glutamicum* is disadvantageous for isolating CGP from the cells in comparison to other microorganisms (Wiefel et al. 2019a).

In the early 2000s, CGP synthesis in *C. glutamicum* was studied for the first time. At that time only a CGP yield of less than 3% of the dry cell mass was obtained from *C. glutamicum* cells (Aboulmagd et al. 2001b). At that time the existence of water-soluble CGP, which usually contains a high proportion of lysine, was not known, yet. For this reason, research on this topic was recently resumed. During this re-evaluation by Wiefel et al. (2019a), different vectors, CphAs and media were examined. It was shown that the strains can accumulate up to 17% of their cells dry mass CGP. It was also shown for the first time that glutamic acid can replace aspartic acid in the backbone of CGP (Wiefel et al. 2019a). The aim of this study was to improve the CGP synthesis in *C. glutamicum* with regard to the incorporation of alternative amino acids into the polymer. For this, optimized cultivation conditions and modified strains of *C. glutamicum* were used to try to increase the proportion of alternative amino acids and to further increase the CGP yield. Two variants of CphA from *Synechocystis sp*. PCC6308 and CphA from *D.* *hafniense* DSM 10664 were used for the investigations. Besides the wild type, mutants of *C. glutamicum* were used, which produce increased amounts of lysine, ornithine or cadaverine.

## Materials and methods

### Bacterial strains, media, and growth conditions

All bacterial stains and plasmids used in this study are listed in Table 1. *E. coli* strains were used for plasmid maintenance and propagation and were cultivated at 30 °C and a stirring rate of 130 rpm in lysogeny broth (LB) medium (Sambrook et al. 1989). Usually, 10 ml medium in 100 ml Erlenmeyer flask were used. For *C. glutamicum* strains BHIS (Tauch et al. 2002), CASO + G (Persicke et al. 2011) and CGXII (Keilhauer et al. 1993) medium were used. Strains harbouring plasmids were grown in presence of 25 μg kanamycin per ml. For the arginine auxotrophic ORN2(P_*gdh*4_) strain (Jensen et al. 2015), 15 mM arginine was added to the CGXII medium. To monitor cell growth, the optical density was measured at 600 nm (OD_600_). For cyanophycin synthesis in *C. glutamicum* two precultures with 10 and 50 ml medium in 100 and 250 ml Erlenmeyer flasks, respectively, were used to inoculate the main culture containing 100 ml in a 500 ml Erlenmeyer flask with two baffles. The first preculture with CASO + G medium was inoculated with a cryoculture and was cultivated for about 8 h. Before inoculating the second preculture, the cells were washed with washing buffer (50 mM TRIS, 50 mM NaCl, pH 6,3). For the second preculture, CGXII medium was used as the main culture. The main culture was inoculated by a 16-h second preculture to an initial OD_600_ of 0.2 and induced after 4 h with 1 mM IPTG. In contrast to the previous studies of Wiefel et al. (2019a) the strains were not cultivated under biotin limiting conditions.

**Table 1:** Strains and plasmids used in this study

Strain or plasmid	Relevant characteristics	Reference or source
Strains		
* E. coli* TOP10	F- *mcrA* Δ(*mrr-hsd* RMS-*mcr*BC) Φ80*lac*ZΔM15 Δ*lac*X74 *rec*A1 *ara*D139 Δ(*araleu*)7697 *ga*/U *ga*/ K *rps*L (Str^R^) *end*A1 *nup*G	Invitrogen, Carlsbad, USA
* C. glutamicum* ATCC 13,032	Wild type, Biotin-auxotroph	DSMZ, Braunschweig, Germany
* C. glutamicum* DM1729	ATCC 13032 *pyc* ^P458S^, *hom* ^V59A^, *lysC* ^T311I^	Georgi et al. (2005)
* C. glutamicum* ORN2(P_*gdh* 4_)	ATCC 13032 with in-frame deletion of prophages CGP1 (cg1507-cg1524), CGP2 (cg1746-cg1752), and CGP3 (cg1890-cg2071), Δ*argFRG*, P_*gdh*4_: − 35: TTGCCA − 10: TATAAT	Jensen et al. (2015)
Plasmids		
pVWEx1	P_tac_, *lacI* ^q^, Km^R^	Peters-Wendisch et al. (2001)
pVWEx1::*cphA*_6308_ Δ1	pVWEx1 with *cphA*_6308_ Δ1 (PstI/XbaI)	Wiefel et al. (2019a)
pVWEx1::*cphA*_6308_ Δ1_C595S	pVWEx1 with *cphA*_6308_ Δ1_C595S (PstI/XbaI)	This study
pVWEx1::*cphA*_Dh_	pVWEx1 with *cphA*_Dh_ (SdaI/XbaI)	Wiefel et al. (2019a)
pEC-XT99A::*ldcC*	P_trc_, *lacI* ^q^, pGA1 OriV_C:g_., *ldcC* from *E. coli* K12, Tet^R^	Sgobba et al. (2018)

*Km*^*R*^ kanamycin resistance, *Tet*^*R*^ tetracycline resistance

### Cultivation of *C. glutamicum* strain DM1729 pVWEx1::*cphA*_6308_ Δ1_C595S in a stirred tank reactor

A Biostat UD30 stainless steel reactor (B. Braun Biotech International, Melsungen, Germany) was used for batch cultivation of *C. glutamicum* strain DM1729 harboring pVWEx1::c*phA*_6308_ Δ1_C595S in 20-liter scale. Two fermentations were performed. In the first fermentation normal CGXII medium according to Keilhauer 1993 was used, only without addition of MOPS buffer. For the second fermentation, 5 g/l CASO broth (Carl Roth, Karlsruhe, Germany) was added to the medium instead of urea. The fermentations were inoculated with an OD_600_ between 0.7 and 0.8. An IPTG concentration of 0.1 mM was used for induction. Cultivation was carried out at 30 °C and a pO_2_ saturation of 30% in the medium, which was controlled by stirring rates between 100 and 800 rpm and aeration rates between 0.5 and 2.0 vvm (volume per volume × minute). The pH was kept constant at a value of 7 with 4 M NaOH or 4 M HCl. As antifoam agent Struktol® J 673 A (Schill + Seilacher, Hamburg, Germany) was used. Samples with a volume of 100 ml were taken to determine OD_600_, cells dry masses and CGP content of the cells.

### Molecular biology techniques

Plasmid DNA from *E. coli* strains was isolated using the GenJET plasmid miniprep kit (Thermo Scientific, Waltham, USA). The plasmids contain the genes for the cyanophycin synthetases CphA_6308_Δ1, CphA_6308_Δ1_C595S or CphA_Dh_ (see Table 2). Upstream of the genes a ribosome binding site (RBS) is located which was optimized for expression in *C. glutamicum* (GAAAGGAGGCCCTTCAG, Siebert and Wendisch 2015). For isolation of plasmids from *C. glutamicum* strains, 15 mg lysozyme were added per ml to the resuspension buffer, and the resuspended cells were incubated for 2 h at 37 °C before following the protocol of the manufacturer (Thermo Scientific, Waltham, USA).

**Table 2:** Details about the used cyanophycin synthetases

CphA	Length	Origin	CGP solubility	Reference
CphA_6308_ Δ1	873 AS	CphA from *Synechocystis* sp. strain PCC 6308 shortened by one AS at the C-terminus	Insoluble and soluble	Steinle et al. (2010)
CphA_6308_ Δ1_C595S	873 AS	CphA from *Synechocystis* sp. strain PCC 6308 with point mutation and shortened by one AS at the C-terminus (see Fig. S1)	Insoluble and soluble	Steinle et al. (2010)
CphA_Dh_	885 AS	CphA from *Desulfitobacterium hafniense* DSM 10664	Only soluble	Ziegler et al. (2002)

Ampicillin was added to the medium during the production of electrocompetent *C. glutamicum* cells (Jensen et al. 2015). Transformation of *C. glutamicum* was performed by electroporation and a heat-shock at 46 °C for 6 min (van der Rest et al. 1999). Restriction analyses were carried out for control purposes. The restriction enzymes were purchased from Thermo Scientific.

### Isolation of cyanophycin from *C. glutamicum*

For isolation of CGP from *C. glutamicum*, cells were harvested by centrifugation (20 min, 5000×*g*, 4 °C), washed with 0.9% (wt/vol) NaCl, and freeze-dried. After determining the cells dry mass, the cell disintegration of the cells was carried out using an ultrasonic disintegrator. For this purpose, the freeze-dried cells were crushed and resuspended in H_2_O (20 ml/g). The suspension was then sonicated for 1 min/ml with the UP200S ultrasonic processor and shaken overnight at 4 °C. Insoluble material was sedimented by centrifugation (30 min, 5000×*g*), and the water-soluble CGP, which was contained in the supernatant, was precipitated using 1.5 vol ice-cold ethanol. After centrifugation, the CGP pellet was washed with acetone and dried at 70 °C. The CGP was dissolved in H_2_O and precipitated again. Water-insoluble CGP was isolated from cell debris by resuspension in 0.1 M HCl. The now dissolved CGP was separated from insoluble material by centrifugation (30 min, 5000×*g*), and it was subsequently precipitated from the supernatant by neutralization (pH 7) using 0.1 to 4 M NaOH. From the supernatant of the insoluble CGP again soluble CGP was isolated. The washing steps were repeated three times for insoluble CGP to further purify the polymer. The precipitated insoluble CGP was then again sedimented by centrifugation and washed twice with H_2_O. Persistent protein impurities were removed after the first precipitation by adding 100 μg proteinase K per ml and by incubating the CGP for 3 h at 60 °C.

### Analysis of isolated cyanophycin

The purity and the molecular masses of isolated CGP were determined by sodium dodecylsulphate polyacrylamide gel electrophoresis (SDS-PAGE) in 11.5% (wt/vol) polyacrylamide gels, according to Laemmli (1970). The apparent molecular weight was estimated using the PageRuler Prestained Protein Ladder (Thermo Scientific).

The amino acid compositions of CGP were determined by high-performance liquid chromatography (HPLC) using a Waters B801 column (300 × 4 mm) after the polymer was converted into derivatives of the constituents as described by Aboulmagd (2001b) and Steinle (2009). Pre-column *ortho*-phthaldialdehyde (OPA) derivatization was performed using a Smartline autosampler 3900 according to the manual (Knauer GmbH, Berlin, Germany). A reference kit (Kollektion AS-10 from Serva Feinbiochemica, Heidelberg, Germany) was used for calibration. Ornithine and citrulline were purchased as monohydrochloride from Fluka or Merck, respectively. CGP was hydrolyzed in 6 M HCl (100 μl/mg) at 95 °C overnight, neutralized, and lyophilized before measurement.

## Results

### Cyanophycin synthesis in *C. glutamicum* ATCC 13032

The CGP synthesis in the wild type of *C. glutamicum* was studied with three different CphAs (CphA_6308_Δ1, CphA_6308_Δ1_C595S and CphA_Dh_). For CGP synthesis the strains were cultivated for 72 h in CGXII medium. Optimizations in cultivation conditions improved cell growth when compared to the previous study of Wiefel et al. (2019a). As a result, the cell density could be increased up to 16.6 g dry mass (DW)/l (see Table 3 and Additional file 1: Fig. S2). All three strains grew similarly well, but in terms of CGP content there were marked differences between the cells harboring different genes for CphAs. With CphA_Dh_ a CGP content over 36% of the cells dry mass was achieved, which corresponds to a CGP concentration of about 6 g/l. In contrast, cells expressing CphA_6308_Δ1 and CphA_6308_Δ1_C595S synthesized significantly less CGP, but the CGP accumulated contained a higher amount of alternative amino acids. The point mutation CphA_6308_Δ1_C595S led to a 4.9 mol% higher lysine content in the insoluble CGP. The lysine content in the CGP of CphA_6308_Δ1_C595S was 13.6 mol% and therefore almost twice as high as that of CphA_Dh_.

**Table 3:** Comparison of different *C. glutamicum* strains for CGP synthesis

Strains	DW [g/l]	Cyanophycin							
		Solubility	CGP content [%DW]	Amino acid composition[mol%] *					
				Asp	Arg	Lys	Glu	Orn	Cit
ATCC 13032									
pVWEx1::*cphA*_6308_Δ1	15.9 ± 0.1	Insoluble	1.9 ± 0.5	50.1	38.9	8.7	0.5	1.0	0.8
		Soluble	4.0 ± 0.1	51.9	26.9	10.9	1.2	6.6	2.5
pVWEx1::*cphA*_6308_Δ1_C595S	15.6 ± 0.2	Insoluble	1.4 ± 0.3	54.7	30.0	13.6	0.3	0.8	0.6
		Soluble	8.3 ± 2.9	51.3	27.5	10.7	0.9	6.4	3.2
pVWEx1::*cphA*_Dh_	16.6 ± 0.4	Insoluble	0.0 ± 0.0	–	–	–	–	–	–
		Soluble	36.2 ± 0.7	49.3	40.1	6.9	2.3	0.6	0.8
DM1279									
pVWEx1::*cphA*_6308_Δ1	11.6 ± 0.3	Insoluble	12.2 ± 2.1	52.9	30.3	16.2	0.2	0.0	0.4
		Soluble	2.9 ± 0.6	45.6	21.0	29.7	1.0	2.2	0.5
pVWEx1::*cphA*_6308_Δ1_C595S	11.7 ± 0.2	Insoluble	13.1 ± 2.3	48.0	29.8	21.6	0.2	0.0	0.4
		Soluble	2.1 ± 0.5	42.7	19.8	33.5	1.0	2.6	0.4
pVWEx1::*cphA*_Dh_	14.4 ± 0.6	Insoluble	0.0 ± 0.0	–	–	–	–	–	–
		Soluble	24.2 ± 1.9	48.3	33.3	15.4	2.2	0.8	0.0
ORN2(P_*gdh*4_)									
pVWEx1::*cphA*_6308_Δ1	11.6 ± 0.2	Insoluble	1.9 ± 0.2	47.6	42.7	0.0	0.3	9.1	0.3
		Soluble	3.2 ± 0.7	46.0	43.5	0.0	0.6	9.4	0.5
pVWEx1::*cphA*_6308_Δ1_C595S	11.7 ± 0.1	Insoluble	2.4 ± 0.2	42.8	47.9	0.0	0.3	8.7	0.3
		Soluble	2.3 ± 0.7	41.7	44.2	0.0	0.7	12.6	0.8
pVWEx1::*cphA*_Dh_	12.9 ± 0.3	Insoluble	0.0 ± 0.0	–	–	–	–	–	–
		Soluble	18.6 ± 1.6	42.8	43.7	0.0	1.4	11.7	0.4

The strains were cultivated for 72 h in 100 ml CGXII medium in a 500 ml Erlenmeyer flask with two baffles at 130 rpm and 30 °C. The cultures were inoculated with an OD_600_ of about 0.2 and after 4 h IPTG was added. For the ORN2(P_*gdh*4_) strains an additional 15 mM arginine was added to the medium. The cells dry mass was determined, and the contained cyanophycin was isolated and analysed. The values indicate the mean and standard deviation of at least three independent cultivations

^*^ The values have been rounded to one decimal place

**Fig. 1:**
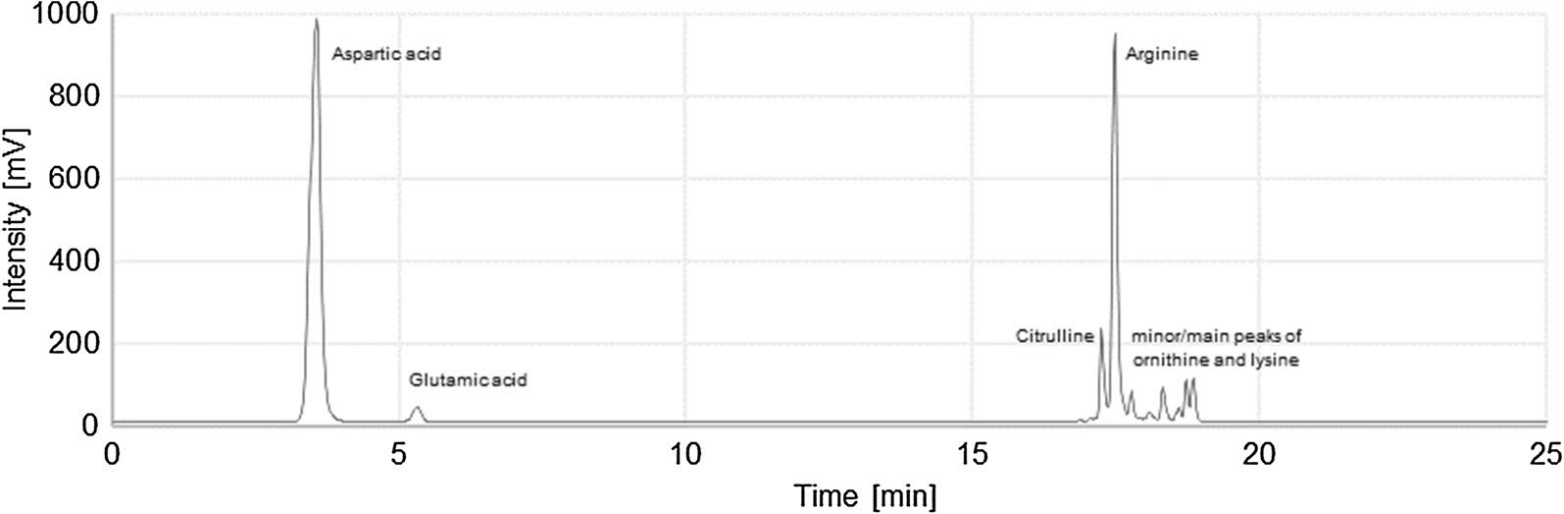
HPLC chromatogram of hydrolysed soluble cyanophycin from *C. glutamicum* ATCC 13032 pVWEx1::*cphA*_6308_Δ1_C595S with all known alternative amino acids

The non-biotin limiting growth conditions in this study had an impact on the composition of the synthesized CGP. The alternative amino acids ornithine and citrulline were found for the first time in the CGP accumulated in *C. glutamicum*. These amino acids were mainly found in the soluble CGPs accumulated in cells expressing CphA_6308_Δ1 or CphA_6308_Δ1_C595S, respectively. This CGP thus contains all the alternative amino acids known to date that are used for in vivo CGP synthesis (see Fig. 1).

### Cyanophycin synthesis in a *C. glutamicum* mutant for lysine production

To increase the lysine content in CGP, CGP synthesis was investigated in *C. glutamicum* strain DM1729 (Georgi et al. 2005), which was developed for the production of lysine. Although the strains did not reach as high cell densities as the cells of *C. glutamicum* ATCC 13032, up to three times more lysine was incorporated into the accumulated CGP (see Table 3 and Additional file 1: Fig. S2). It is remarkable that the strain grew better with the CphA_Dh_ and reached a higher CGP content with 24.2% of cells dry mass. The lysine content in the CGP was 15.4 mol% and was therefore significantly higher than in the CGP from the cells of *C.* *glutamicum* ATCC 13032 pVWEx1::*cphA*_Dh_. With the other two CphAs, lysine contents of up to 33.5 mol% were measured in the soluble CGP, and even in the insoluble CGP higher lysine contents were found. The higher lysine concentrations in the cells of these strains resulted in a strong increase in insoluble CGP with up to 13.1% of cells dry mass at a lysine content of up to 21.6 mol%. It was previously thought that the higher lysine concentration would have a greater effect on soluble CGP, as high lysine is often a characteristic of soluble CGP (Frommeyer and Steinbüchel 2013). The insoluble CGP synthesised with CphA_6308_Δ1_C595S showed a 5.4 mol% higher lysine content in comparison to the CGP accumulated by the single mutant cyanophycin synthetase. The content of ornithine and citrulline was lower in all these strains than in the *C. glutamicum* ATCC 13032 strains.

### Cyanophycin synthesis in a *C. glutamicum* mutant for ornithine production

*Corynebacterium glutamicum* ORN2(P_*gdh*4_) is a strain developed for the increased production of ornithine (Jensen et al. 2015). In this strain, genes of the arginine biosynthesis pathway were deleted to prevent the further metabolism of ornithine. As a result, the strain is auxotrophic for arginine. In a preliminary experiment it was shown that a concentration of 15 mM arginine in the CGXII medium is a suitable concentration for CGP synthesis, as this concentration allows good growth.

**Fig. 2:**
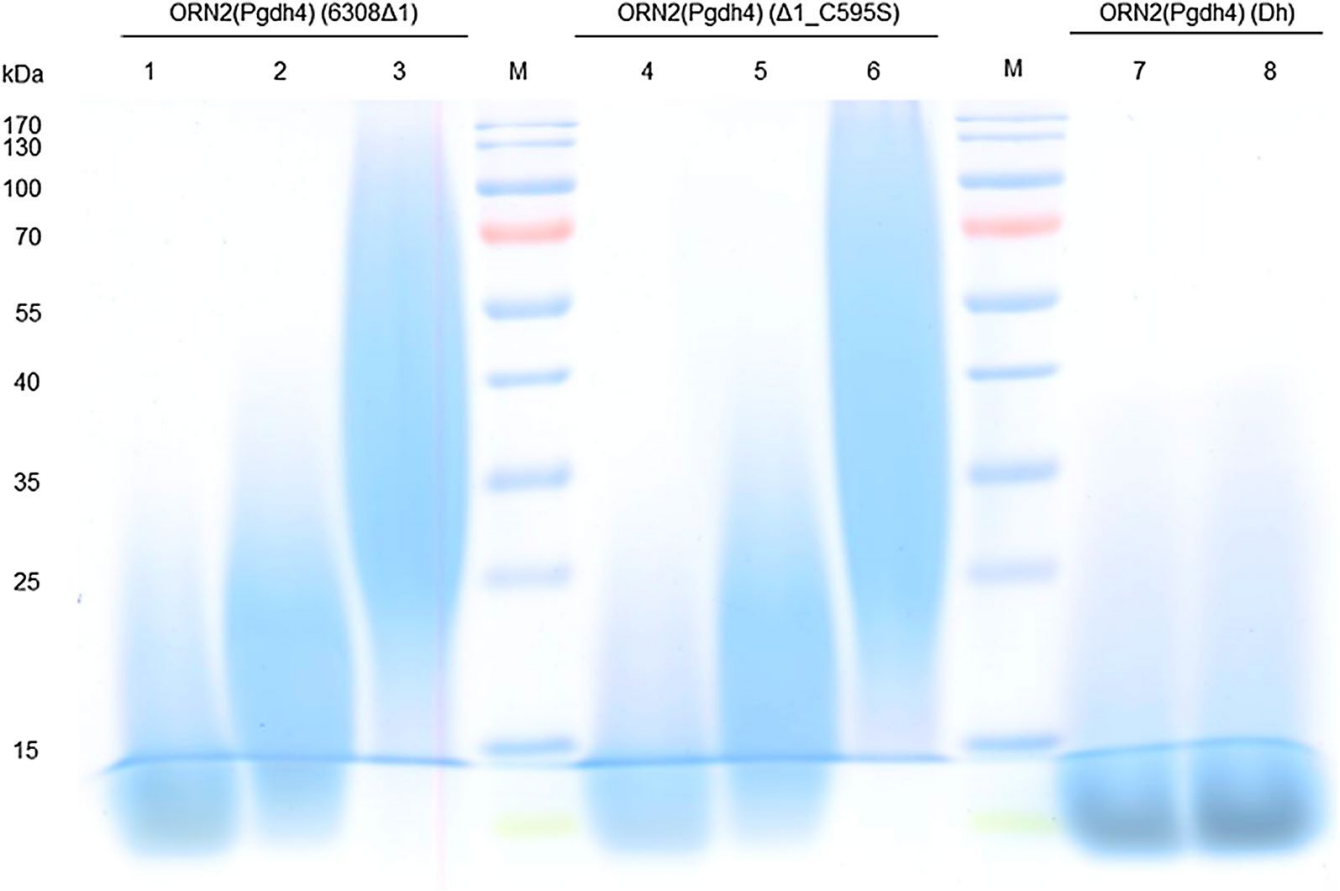
SDS-PAGE analysis of CGP synthesized in *C. glutamicum* ORN2(P_*gdh*4_) expressing different CphAs when cultivated in CGXII medium with 15 mM arginine. The SDS-PAGE shows the separation of 100 μg of soluble (1, 2, 4, 5, 7 and 8) and insoluble (3 and 6) CGP from *C. glutamicum* ORN2(P_*gdh*4_) pVWEx1::*cphA*_6308_Δ1, pVWEx1::*cphA*_6308_Δ1_C595S and pVWEx1::*cphA*_Dh_, compared to a molecular mass marker (M)

The CGP content of these strains is nevertheless lower than that of the other *C. glutamicum* strains investigated. With these strains ornithine contents of 10 mol% on average were achieved (see Table 3 and Additional file 1: Fig. S2). These results represent a slight improvement when compared to the previous results with *Saccharomyces cerevisiae*, where ornithine contents of only 8 mol% were achieved (Steinle et al. 2009). There were only slight differences in the amino acid composition between the different isolates. The SDS-PAGE analysis showed that there are large differences in the molecular masses of the accumulated soluble and insoluble CGPs (see Fig. 2). Most of the soluble CGP exhibited molecular masses below 15 kDa (lane 1, 4, 7 and 8). In the case of CphA_Dh_ it is clearly visible that most CGP has a molecular mass of only 10 kDa. The soluble CGP isolated from the supernatant of the insoluble CGP is slightly larger with 15 to 25 kDa (lane 2 and 5). The insoluble CGP, on the other hand, exhibited a molecular weight of 35 to 55 kDa (lane 3 and 6).

### Cyanophycin synthesis in a *C. glutamicum* mutant for cadaverine production

In a previous in vitro study, it was shown that agmatine (biogenic amine of arginine) has a negative effect on the incorporation of arginine into the polymer. Therefore, it was suspected that it could be incorporated into CGP instead of arginine (Aboulmagd 2001a). Cadaverine is also a biogenic amine and is produced by decarboxylation of lysine. To enable the synthesis of cadaverine in *C. glutamicum*, the lysine decarboxylase LdcC was additionally introduced into the DM1729 strains using the vector pEC-XT99A (Sgobba et al. 2018). This is the first study on the effect of cadaverine on CGP synthesis. The conversion of lysine to cadaverine results in a significant decrease in the lysine content of CGP (see Table 4 and Additional file 1: Fig. S2). The CGP content in the cells also decreases in comparison to the DM1729 strains not harboring pEC-XT99A::*ldcC*. Noticeable was the slower growth of the strain expressing CphA_Dh_. Cadaverine could not be detected in any of the extracted CGP. Possibly cadaverine has an inhibiting effect on CGP synthesis.

**Table 4:** Comparison of CGP synthesis in *C. glutamicum* strains for cadaverine production

Strains	DW [g/l]	Cyanophycin								
		Solubility	CGP content [%DW]	Amino acid composition [mol%] *						
				Asp	Arg	Lys	Glu	Orn	Cit	Cad
DM1279 pEC-XT99A::*ldcC*										
pVWEx1::*cphA*_6308_Δ1	13.3 ± 0.1	Insoluble	3.5 ± 0.9	46.6	46.2	6.2	0.4	0.1	0.5	0.0
		Soluble	3.5 ± 1.4	44.1	38.0	13.6	1.6	2.2	0.5	0.0
pVWEx1::*cphA*_6308_Δ1_C595S	12.7 ± 0.3	Insoluble	3.2 ± 1.2	47.4	45.8	5.9	0.4	0.1	0.4	0.0
		Soluble	1.5 ± 1.2	44.2	37.1	16.0	1.9	0.0	0.8	0.0
pVWEx1::*cphA*_Dh_	9.2 ± 0.1	Insoluble	0.0 ± 0.0	–	–	–	–	–	–	–
		Soluble	20.4 ± 0.7	44.1	41.9	11.0	2.2	0.5	0.3	0.0

The strains were cultivated for 72 h in 100 ml CGXII medium in 500 ml Erlenmeyer flasks with two baffles at 130 rpm and 30 °C. The cultures were inoculated with an OD_600_ of about 0.2 and after 4 h IPTG was added. The cells dry mass was determined and the contained cyanophycin was isolated and analyzed. The values indicate the mean and standard deviation of at least three independent cultivations

^*^ The values have been rounded to one decimal place

### Cultivation in stirred tank reactors and scale up

In the first fermentation of *C. glutamicum* DM1729 pVWEx1::*cphA*_6308_ Δ1_C595S in CGXII medium CGP biosynthesis was induced with 0.1 mM IPTG after a cultivation period of 4 h. The fermentation took 53 h in total. A maximum cells dry mass of 11 g/l was obtained. Only insoluble CGP could be isolated. This reached an average of 2.5% of the cells dry mass. The lysine content was 16.7 mol%.

In the second fermentation with addition of 5 g/l CASO broth, 0.1 mM IPTG was used directly at the start of the fermentation to induce cells for production of CGP. Based on the OD_600_, it could be seen that the cells grew significantly faster and they reached a higher cells dry mass of 15 g/l than in the flask. Although soluble and insoluble CGP could be isolated, the total CGP content was again only 2.5% of the cells dry mass. The amino acid composition was comparable to that of the CGP formed in the flask experiment.

## Discussion

Based on the study of Wiefel et al. (2019a), the cultivation conditions were further optimized to improve cell growth and to increase CGP yield. The omission of biotin limitation had the greatest impact, even if this means that less glutamic acid is incorporated into CGP. The growth of the wild type strains could thus be increased to a cells dry mass of 16 g/l, and a CGP content of 36.2% of the cells dry mass was achieved with CphA_Dh_. In comparison, the cultivation of *C. glutamicum* ATCC 13032 pVWEx1::*cphA*_Dh_ with biotin limitation in Wiefel et al. (2019a) achieved only a CGP content of 17.1% of cells dry mass. For the CphA variants of *Synechocystis sp.* PCC6308 it was possible for the first time to isolate water soluble CGP from cells cultivated in CGXII medium. The CGP contents of cells harboring these CphAs were significantly lower than that of cells harboring CphA_Dh_. However, due to the soluble CGP, a slight increase compared to previous studies (Aboulmagd et al. 2001b; Wiefel et al. 2019a) was observed. Due to the changes in cultivation conditions, ornithine and citrulline could be detected in CGP from *C. glutamicum* for the first time. However, the amounts of citrulline were very low. With *S. cerevisiae*, for example, citrulline contents of 20 mol% have been already achieved (Steinle et al. 2009).

Experiments with the mutants of *C. glutamicum* showed that the proportion of alternative amino acids in CGP can be increased in these strains. The lysine contents were more than doubled by DM1729 in many samples and much more insoluble CGP was synthesized. This could possibly be related to the intracellular pH increasing due to the higher lysine concentration and thus being closer to the optimal pH of 8.2 of CphA_6308_ (Aboulmagd et al. 2001a). It is noticeable that due to the point mutation C595S in the insoluble CGP the lysine content is 5.4 mol% higher. Also in *P. pastoris* and *E. coli* the lysine contents were slightly higher if the point mutation C595S of CphA was used (Steinle et al. 2010). However, the increase was not as high as in this case. This effect has already been observed with *C. glutamicum* ATCC 13032. The point mutation seems to have a positive influence on the incorporation of lysine.

An increase of the alternative amino acid was also observed in the ornithine-forming strains. Here, a maximum ornithine content of 12.6 mol% was reached. The proportion is thus higher than in the study of Steinle et al. 2009, where with *S. cerevisiae* an ornithine proportion of 8 mol% was achieved. The differences in the composition of soluble and insoluble CGP were very small in these strains, which suggests that the size of the polymer also has an influence on the solubility behavior, as this was the main difference. This assumption was already made in Wiefel et al. (2019a).

The decarboxylation of lysine to cadaverine has resulted in a lower cellular cyanophycin content and thus in less CGP synthesised and in a drastic reduction in the lysine content of the polymer. However, no cadaverine could be detected in cyanophycin. The strains for cadaverine production have therefore proved unsuitable for cyanophycin synthesis.

Although the growth of the mutants is lower than that of *C. glutamicum* ATCC 13032, mutants that produce more lysine in particular have shown great potential for CGP synthesis. It would therefore be useful to investigate this further and to optimize the scale up, because until now growth has been improved on a larger scale, but the CGP content is lower than in flasks.

## Supplementary Information

**Additional file 1.**: **Fig. S1**. Sequence alignment of *cph*A_6308_ and *cph*A_6308_Δ1_C595S. **Fig. S2**. Graphical representation of the results obtained with the different *C. glutamicum* strains for CGP synthesis.

## Data Availability

All datasets on which the conclusions of the manuscript are presented in the main paper.

## Abbreviations

Arg: Arginine
AS: Amino acids
Asp: Aspartic acid
Cad: Cadaverine
CGP: Cyanophycin grana peptide
Cit: Citrulline
CphA: Cyanophycin synthetases
CphB: Intracellular cyanophycinase
CphE: Extracellular cyanophycinase
DW: Dry weight
Glu: Glutamic acid
LB: Lysogeny broth
Lys: Lysine
OD_600_: Optical density at 600 nm
OPA: *ortho*-Phthaldialdehyde
Orn: Ornithine
RBS: Ribosome binding site
WT: Wild type
