# Supplementary material for: Incorporation of alternative amino acids into cyanophycin by different cyanophycin synthetases heterologously expressed in Corynebacterium glutamicum
Source: AMB Express. 2021 Apr 15;11:55. doi: 10.1186/s13568-021-01217-5 (PMC8050183; doi:10.1186/s13568-021-01217-5)
Supplement: Supplementary file 1 — Additional file 1.: Fig. S1. Sequence alignment of cphA6308 and cphA6308Δ1_C595S. Fig. S2. Graphical representation of the results obtained with the different C. glutamicum strains for CGP synthesis. [file 13568_2021_1217_MOESM1_ESM.docx]

**Additional file 1**

| *cphA*_6308_  *cphA*_6308_Δ1_C595S | 1  1 | ATGAAAATCCTCAAAACACAAACCCTACGGGGACCTAACTATTGGAGTATCCGTCGCCAA  ATGAAAATCCTCAAAACACAAACCCTACGGGGACCTAACTATTGGAGTATCCGTCGCCAA | 60  60 |
| --- | --- | --- | --- |
| *cphA*_6308_  *cphA*_6308_Δ1_C595S | 61  61 | AAATTAATCCAAATGCGTCTGGATTTAGAAGACGTAGCCGAAAAACCTTCTAACCTGATA  AAATTAATCCAAATGCGTCTGGATTTAGAAGACGTAGCCGAAAAACCTTCTAACCTGATA | 120  120 |
| *cphA*_6308_  *cphA*_6308_Δ1_C595S | 121  121 | CCGGGGTTTTATGAAGGCTTAGTGAAAATATTACCCTCTTTAGTAGAACATTTCTGTTCC  CCGGGGTTTTATGAAGGCTTAGTGAAAATATTACCCTCTTTAGTAGAACATTTCTGTTCC | 180  180 |
| *cphA*_6308_  *cphA*_6308_Δ1_C595S | 180  180 | AGAGATCATCGAGGAGGCTTCCTAGAAAGAGTACAAGAAGGTACTTATATGGGGCATATC  AGAGATCATCGAGGAGGCTTCCTAGAAAGAGTACAAGAAGGTACTTATATGGGGCATATC | 240  240 |
| *cphA*_6308_  *cphA*_6308_Δ1_C595S | 241  241 | ATTGAACACATTGCCCTAGAATTGCAAGAATTAGCAGGAATGCCTGTAGGTTTTGGGCGT  ATTGAACACATTGCCCTAGAATTGCAAGAATTAGCAGGAATGCCTGTAGGTTTTGGGCGT | 300  300 |
| *cphA*_6308_  *cphA*_6308_Δ1_C595S | 301  301 | ACCAGAGAAACTTCAACTCCGGGTATTTATAACGTAGTTTTTGAGTATGTTTATGAGGAG  ACCAGAGAAACTTCAACTCCGGGTATTTATAACGTAGTTTTTGAGTATGTTTATGAGGAG | 360  360 |
| *cphA*_6308_  *cphA*_6308_Δ1_C595S | 361  361 | GCTGGAAGATACGCAGGTAGAGCCGCCGTCAGACTTTGTAACTCTATCATTACTACAGGG  GCTGGAAGATACGCAGGTAGAGCCGCCGTCAGACTTTGTAACTCTATCATTACTACAGGG | 420  420 |
| *cphA*_6308_  *cphA*_6308_Δ1_C595S | 421  421 | GCTTATGGGCTAGACGAATTAGCTCAAGATTTATCTGATCTCAAGGATTTACGAGCCAAT  GCTTATGGGCTAGACGAATTAGCTCAAGATTTATCTGATCTCAAGGATTTACGAGCCAAT | 480  480 |
| *cphA*_6308_  *cphA*_6308_Δ1_C595S | 481  481 | TCTGCTTTAGGTCCTTCCACCGAAACTATTATCAAGGAAGCCGAAGCGAGACAAATTCCT  TCTGCTTTAGGTCCTTCCACCGAAACTATTATCAAGGAAGCCGAAGCGAGACAAATTCCT | 540  540 |
| *cphA*_6308_  *cphA*_6308_Δ1_C595S | 541  541 | TGGATGTTGCTTAGTGCAAGGGCAATGGTACAGTTAGGTTATGGGGCAAATCAACAACGG  TGGATGTTGCTTAGTGCAAGGGCAATGGTACAGTTAGGTTATGGGGCAAATCAACAACGG | 600  600 |
| *cphA*_6308_  *cphA*_6308_Δ1_C595S | 601  601 | ATTCAAGCAACTCTGAGTAATAAAACGGGCATCTTAGGGGTGGAGTTAGCTTGTGATAAA  ATTCAAGCAACTCTGAGTAATAAAACGGGCATCTTAGGGGTGGAGTTAGCTTGTGATAAA | 660  660 |
| *cphA*_6308_  *cphA*_6308_Δ1_C595S | 661  661 | GAAGGCACTAAAACCACTTTAGCGGAAGCTGGTATTCCTGTGCCTAGGGGGACTGTTATT  GAAGGCACTAAAACCACTTTAGCGGAAGCTGGTATTCCTGTGCCTAGGGGGACTGTTATT | 720  720 |
| *cphA*_6308_  *cphA*_6308_Δ1_C595S | 721  721 | TACTATGCTGATGAATTAGCTGATGCCATCGCTGATGTCGGTGGTTATCCCATTGTTCTC  TACTATGCTGATGAATTAGCTGATGCCATCGCTGATGTCGGTGGTTATCCCATTGTTCTC | 780  780 |
| *cphA*_6308_  *cphA*_6308_Δ1_C595S | 781  781 | AAACCCTTAGACGGCAATCATGGTAGAGGCATTACCATTGATATTAACTCTCAACAGGAA  AAACCCTTAGACGGCAATCATGGTAGAGGCATTACCATTGATATTAACTCTCAACAGGAA | 840  840 |
| *cphA*_6308_  *cphA*_6308_Δ1_C595S | 841  841 | GCTGAGGAGGCTTATGATTTGGCTAGTGCCGCTTCTAAAACTCGATCAGTCATTGTGGAA  GCTGAGGAGGCTTATGATTTGGCTAGTGCCGCTTCTAAAACTCGATCAGTCATTGTGGAA | 900  900 |
| *cphA*_6308_  *cphA*_6308_Δ1_C595S | 901  901 | AGATACTATAAAGGTAATGATCATCGGGTTTTAGTCATCAATGGTAAACTGGTGGCAGTC  AGATACTATAAAGGTAATGATCATCGGGTTTTAGTCATCAATGGTAAACTGGTGGCAGTC | 960  960 |
| *cphA*_6308_  *cphA*_6308_Δ1_C595S | 961  961 | TCCGAGAGAATCCCCGCCCACGTCACGGGCAATGGTTCTTCTACCATTGAGGAATTGATT  TCCGAGAGAATCCCCGCCCACGTCACGGGCAATGGTTCTTCTACCATTGAGGAATTGATT | 1020  1020 |
| *cphA*_6308_  *cphA*_6308_Δ1_C595S | 1021  1021 | CAAGAAACCAATGAACATCCCGATCGAGGTGACGGTCATGATAATGTCTTAACTAGAATC  CAAGAAACCAATGAACATCCCGATCGAGGTGACGGTCATGATAATGTCTTAACTAGAATC | 1080  1080 |
| *cphA*_6308_  *cphA*_6308_Δ1_C595S | 1081  1081 | AGTATCGATCGAACTTCCTTAGGAGTCTTAAAAAGACAAGGTTTCGAGATGGATACCGTT  AGTATCGATCGAACTTCCTTAGGAGTCTTAAAAAGACAAGGTTTCGAGATGGATACCGTT | 1140  1140 |
| *cphA*_6308_  *cphA*_6308_Δ1_C595S | 1141  1141 | CTCAAAAAAGGAGAAGTTGCCTATTTACGAGCTACGGCTAACCTTAGTACCGGGGGTATT  CTCAAAAAAGGAGAAGTTGCCTATTTACGAGCTACGGCTAACCTTAGTACCGGGGGTATT | 1200  1200 |
| *cphA*_6308_  *cphA*_6308_Δ1_C595S | 1201  1201 | GCCATCGATCGAACCGATGAAATACATCCTCAAAATATTTGGATAGCCGAAAGAGTCGCC  GCCATCGATCGAACCGATGAAATACATCCTCAAAATATTTGGATAGCCGAAAGAGTCGCC | 1260  1260 |
| *cphA*_6308_  *cphA*_6308_Δ1_C595S | 1261  1261 | AAAATTATTGGTTTAGACATTGCTGGAATTGACGTAGTTACCCCAGACATTACCAAACCC  AAAATTATTGGTTTAGACATTGCTGGAATTGACGTAGTTACCCCAGACATTACCAAACCC | 1320  1320 |
| *cphA*_6308_  *cphA*_6308_Δ1_C595S | 1321  1321 | TTAACAGAAGTCGATGGGGTAATTGTGGAAGTCAACGCTGCCCCCGGCTTTAGGATGCAC  TTAACAGAAGTCGATGGGGTAATTGTGGAAGTCAACGCTGCCCCCGGCTTTAGGATGCAC | 1380  1380 |
| *cphA*_6308_  *cphA*_6308_Δ1_C595S | 1381  1381 | GTTGCCCCCAGTCAAGGCTTACCCCGTAACGTAGCCGCTCCCGTCATCGATATGTTATTC  GTTGCCCCCAGTCAAGGCTTACCCCGTAACGTAGCCGCTCCCGTCATCGATATGTTATTC | 1440  1440 |
| *cphA*_6308_  *cphA*_6308_Δ1_C595S | 1441  1441 | CCTGATAATCACCCTAGTCGCATCCCCATTTTAGCGGTGACAGGCACAAACGGAAAAACC  CCTGATAATCACCCTAGTCGCATCCCCATTTTAGCGGTGACAGGCACAAACGGAAAAACC | 1500  1500 |
| *cphA*_6308_  *cphA*_6308_Δ1_C595S | 1501  1501 | ACCACGACTAGACTATTAGCCCATATCTATCGTCAAACGGGGAAAGTTGTGGGTTATACC  ACCACGACTAGACTATTAGCCCATATCTATCGTCAAACGGGGAAAGTTGTGGGTTATACC | 1560  1560 |
| *cphA*_6308_  *cphA*_6308_Δ1_C595S | 1561  1561 | AGTACTGACGGTATTTACTTAGGTGATTACATGGTAGAAAAAGGCGATAACACTGGTCCT  AGTACTGACGGTATTTACTTAGGTGATTACATGGTAGAAAAAGGCGATAACACTGGTCCT | 1620  1620 |
| *cphA*_6308_  *cphA*_6308_Δ1_C595S | 1621  1621 | GTGAGCGCTGGAGTGATTTTGAGAGACCCCACCGTAGAAGTTGCCGTGTTAGAGTGTGCT  GTGAGCGCTGGAGTGATTTTGAGAGACCCCACCGTAGAAGTTGCCGTGTTAGAGTGTGCT | 1680  1680 |
| *cphA*_6308_  *cphA*_6308_Δ1_C595S | 1681  1681 | AGAGGGGGAATTTTACGATCGGGTTTAGCCTTTGAAAGCT**G**TGATGTTGGCGTTGTCTTA  AGAGGGGGAATTTTACGATCGGGTTTAGCCTTTGAAAGCT**C**TGATGTTGGCGTTGTCTTA | 1740  1740 |
| *cphA*_6308_  *cphA*_6308_Δ1_C595S | 1741  1741 | AACGTAGCCGAAGATCATCTAGGATTGGGAGACATAGACACCATCGAACAAATGGCAAAA  AACGTAGCCGAAGATCATCTAGGATTGGGAGACATAGACACCATCGAACAAATGGCAAAA | 1800  1800 |
| *cphA*_6308_  *cphA*_6308_Δ1_C595S | 1801  1801 | GTAAAAGGAGTCATCGCCGAATCAGTCAATGCCGATGGTTATGCAGTCCTGAATGCAGAC  GTAAAAGGAGTCATCGCCGAATCAGTCAATGCCGATGGTTATGCAGTCCTGAATGCAGAC | 1860  1860 |
| *cphA*_6308_  *cphA*_6308_Δ1_C595S | 1861  1861 | GATCCTTTAGTAGCACAAATGGCGAAAAATGTCAAGGGTAAAATTGCTTATTTTTCCATG  GATCCTTTAGTAGCACAAATGGCGAAAAATGTCAAGGGTAAAATTGCTTATTTTTCCATG | 1920  1920 |
| *cphA*_6308_  *cphA*_6308_Δ1_C595S | 1921  1921 | AGTAAAGATAATCCCATCATTATCGATCATTTACGGCGTAATGGTATGGCGGCGGTGTAT  AGTAAAGATAATCCCATCATTATCGATCATTTACGGCGTAATGGTATGGCGGCGGTGTAT | 1960  1960 |
| *cphA*_6308_  *cphA*_6308_Δ1_C595S | 1981  1981 | GAAAACGGCTATCTTTCCATTTTTGAAGGAGAATGGACATTAAGAATTGAGAAAGCGGAA  GAAAACGGCTATCTTTCCATTTTTGAAGGAGAATGGACATTAAGAATTGAGAAAGCGGAA | 2040  2040 |
| *cphA*_6308_  *cphA*_6308_Δ1_C595S | 2041  2041 | AATATCCCTGTTACCATGAAAGCCATGGCGCCTTTTATGATTGCCAATGCTTTAGCCGCA  AATATCCCTGTTACCATGAAAGCCATGGCGCCTTTTATGATTGCCAATGCTTTAGCCGCA | 2100  2100 |
| *cphA*_6308_  *cphA*_6308_Δ1_C595S | 2101  2101 | TCCTTAGCCGCCTTTGTTCACGGTATCGACATCGAATTAATTCGTCAAGGGGTACGCAGT  TCCTTAGCCGCCTTTGTTCACGGTATCGACATCGAATTAATTCGTCAAGGGGTACGCAGT | 2160  2160 |
| *cphA*_6308_  *cphA*_6308_Δ1_C595S | 2161  2161 | TTTAACCCTGGGGCGAATCAAACCCCCGGCAGAATGAACCTTTTTGACATGAAAGATTTT  TTTAACCCTGGGGCGAATCAAACCCCCGGCAGAATGAACCTTTTTGACATGAAAGATTTT | 2220  2220 |
| *cphA*_6308_  *cphA*_6308_Δ1_C595S | 2221  2221 | TCTGTGTTGATCGATTACGCCCATAATCCTGCGGGTTATTTAGCCGTAGGGGAGTTTGTG  TCTGTGTTGATCGATTACGCCCATAATCCTGCGGGTTATTTAGCCGTAGGGGAGTTTGTG | 2280  2280 |
| *cphA*_6308_  *cphA*_6308_Δ1_C595S | 2281  2281 | AAAAATTGGAAGGGCGATCGCCTTGGAGTAATCGGAGGACCGGGTGACAGACGGGATGAG  AAAAATTGGAAGGGCGATCGCCTTGGAGTAATCGGAGGACCGGGTGACAGACGGGATGAG | 2340  2340 |
| *cphA*_6308_  *cphA*_6308_Δ1_C595S | 2341  2341 | GATTTGATGCTTTTAGGTAAAATTGCCTCTCAAATTTTTGATCACATTATCATCAAAGAA  GATTTGATGCTTTTAGGTAAAATTGCCTCTCAAATTTTTGATCACATTATCATCAAAGAA | 2400  2400 |
| *cphA*_6308_  *cphA*_6308_Δ1_C595S | 2401  2401 | GATGACGATAATCGAGGGCGCGATCGAGGTACTGTAGCTGATTTGATAGCTAAAGGTATT  GATGACGATAATCGAGGGCGCGATCGAGGTACTGTAGCTGATTTGATAGCTAAAGGTATT | 2460  2460 |
| *cphA*_6308_  *cphA*_6308_Δ1_C595S | 2461  2461 | GTGGCTGAAAATCCTAACGCTAGTTATGATGTCATCCTCGATGAAACCGAAGCCATTGAA  GTGGCTGAAAATCCTAACGCTAGTTATGATGTCATCCTCGATGAAACCGAAGCCATTGAA | 2520  2520 |
| *cphA*_6308_  *cphA*_6308_Δ1_C595S | 2521  2521 | ACTGGACTCAAAAAAGTCGATAAAGGTGGTTTAGTAGTTATTTTCCCTGAAAGTGTTACA  ACTGGACTCAAAAAAGTCGATAAAGGTGGTTTAGTAGTTATTTTCCCTGAAAGTGTTACA | 2580  2580 |
| *cphA*_6308_  *cphA*_6308_Δ1_C595S | 2581  2581 | GGTTCGATCGAGATGATTGAGAAATATCATCTCAGTAGTGAATAG  GGTTCGATCGAGATGATTGAGAAATATCATCTCAGTAGT - - - TAG | 2625  2622 |

**Fig. S1** Sequence alignment of *cphA*_6308_ and *cphA*_6308_Δ1_C595S

Comparison of the DNA sequences of *cphA*_6308_ from *Synechocystis* sp. strain PCC 6308 and the double mutant *cphA*_6308_Δ1_C595S. The point mutation is actually located at position C574S but is still referred to as C595S because of a designation error in earlier publications (among others in Kroll 2009, Steinle 2010, Kroll 2011, Frommeyer 2014 and Frommeyer 2016).

| **a** | **b** |
| --- | --- |
| **c** | **d** |

Fig. S2 Graphical representation of the results obtained with the different *C. glutamicum* strains for CGP synthesis

a) Comparison of cell dry masses from the different *C. glutamicum* strains with the different CphAs harvested after a 72 h cultivation period. Cultivations were performed at least as triplicate determinations and standard deviations serve as error indicators.

b) Comparison of CGP content as a percentage of cell dry weight in the studied *C. glutamicum* strains. The amount of water-insoluble CGP is shown in dark gray and the water-soluble CGP in light gray.

c) Comparison of amino acid composition of insoluble CGP isolates. Legend: Asp (gray), Glu (black), Cit (red), Arg (blue), Lys (green), and Orn (orange)

d) Comparison of amino acid composition of the soluble CGP isolates. Legend: as in c)

**Supplemental References**

Frommeyer M, Bergander K, Steinbüchel A (2014) Guanidination of soluble lysine-rich cyanophycin yields a homoarginine-containing polyamide. Appl Environ Microbiol 80:2381-2389. https://doi.org/10.1128/AEM.04013-13

Frommeyer M, Wiefel L, Steinbüchel A (2016) Features of the biotechnologically relevant polyamide family “cyanophycins” and their biosynthesis in prokaryotes and eukaryotes. Critical Reviews in Biotechnology 36:153-164. https://doi.org/10.3109/07388551.2014.946467

Kroll J, Klinter S, Steinbüchel A (2011) A novel plasmid addiction system for large-scale production of cyanophycin in *Escherichia coli* using mineral salts medium. Appl Microbiol Biotechnol 89: 593-604. https://doi.org/10.1007/s00253-010-2899-2

Kroll J, Steinle A, Reichelt R, Ewering C, Steinbüchel A (2009). Establishment of a novel anabolism-based addiction system with an artificially introduced mevalonate pathway: complete stabilization of plasmids as universal application in white biotechnology. Metab Eng 11:168-177. https://doi.org/10.1016/j.ymben.2009.01.007

Steinle A, Witthoff S, Krause JP, Steinbüchel A (2010) Establishment of cyanophycin biosynthesis in *Pichia pastoris* and optimization by use of engineered cyanophycin synthetases. Appl Environ Microbiol 76:1062–1070. https://doi.org/10.1128/AEM.01659-09
